# Defining a Standard Set of Patient-Reported Outcomes for Patients With Advanced Ovarian Cancer

**DOI:** 10.3389/fonc.2022.885910

**Published:** 2022-05-18

**Authors:** Vicente Escudero-Vilaplana, Elsa Bernal, Gema Casado, Roberto Collado-Borrell, Raúl Diez-Fernández, Ana Beatriz Fernández Román, Carlos Folguera, Lucía González-Cortijo, Marta Herrero-Fernández, Gloria Marquina, Concepción Martínez Nieto, Miguel Angel Rodríguez, Ana Rosa Rubio, Patricia Sanmartin-Fenollera, Maria José Vazquez Castillo, Marta Comellas, Eva Maria Guerra

**Affiliations:** ^1^Hospital Pharmacy, Hospital General Universitario Gregorio Marañón, Madrid, Spain; ^2^Department of Medical Oncology, Hospital Universitario 12 de Octubre, Madrid, Spain; ^3^Hospital Pharmacy, Hospital Universitario La Paz, Madrid, Spain; ^4^Hospital Pharmacy, Hospital Universitario de Getafe, Getafe, Spain; ^5^Hospital Pharmacy, Hospital Universitario de Fuenlabrada, Madrid, Spain; ^6^Hospital Pharmacy, Hospital Universitario Puerta de Hierro Majadahonda, Majadahonda, Spain; ^7^Breast and Gynecological Tumor Unit, Hospital Universitario Quirónsalud Madrid, Madrid, Spain; ^8^Hospital Pharmacy, Hospital Universitario Príncipe de Asturias, Alcalá de Henares, Spain; ^9^Department of Medical Oncology, Hospital Clinico san Carlos, Madrid, Spain; ^10^Department of Medicine, School of Medicine, Universidad Complutense de Madrid (UCM), Instituto de Investigación Sanitaria del Hospital Clínico San Carlos (IdISSC), Madrid, Spain; ^11^Hospital Pharmacy, Hospital Universitario La Princesa, Madrid, Spain; ^12^Hospital Pharmacy, Hospital Universitario Ramón y Cajal, Madrid, Spain; ^13^Hospital Pharmacy, Hospital Universitario de Toledo, Toledo, Spain; ^14^Hospital Pharmacy, Hospital Universitario Fundación Alcorcón, Alcorcón, Spain; ^15^Hospital Pharmacy, Hospital Universitario de Móstoles, Móstoles, Spain; ^16^Outcomes’10, Castellón de la Plana, Spain; ^17^Department of Medical Oncology, Hospital Universitario Ramón y Cajal, Madrid, Spain

**Keywords:** ovarian cancer, patient-centered care, patient-reported outcomes, patient-reported outcome measures, patient centricity

## Abstract

**Purpose:**

Advanced ovarian cancer (AOC) and its treatment cause several symptoms and impact on patients’ health-related quality of life (HRQoL). We aim to reach a consensus on the most relevant patient-reported outcome (PROs), the corresponding measures (PROMs), and measurement frequency during AOC patients’ follow-up from patients’ and healthcare professionals’ (HCP) perspective.

**Methods:**

The project comprised five steps: 1) a literature review, 2) a focus group with patients, 3) a nominal group with HCP, 4) two round-Delphi consultations with patients and HCP, and 5) a final meeting with HCP. Delphi questionnaire was elaborated based on literature review, focus group (n=5 patients), and nominal group (n=16 HCP). The relevance of each PRO and the appropriateness (A) and feasibility (F) of the proposed PROM were assessed (Likert scale 1=strongly agree; 9=strongly disagree). The consensus was reached when at least 75% of the panelists rated it as ‘relevant’, ‘appropriate’, or ‘feasible’ (score 7-9).

**Results:**

A total of 56 HCP [51.8% Hospital Pharmacy; 41.1% Oncology; 3.6% Nursing; and 3.6% Psycho-oncology; mean time in specialty 12.5 (8.0) years] and 10 AOC patients [mean time diagnosis 5.4 (3.0) years] participated in the 1^st^ round. All PROs achieved consensus regarding their relevance, except dry skin (58.0%). Agreement was reached for PRO-CTCAE to be used to assess fatigue (A:84.9%; F:75.8%), neuropathy (A:92.4%; F:77.3%), diarrhea (A:87.9%; F:88.7%), constipation (A:86.4%; F:75.8%), nausea (A:89.4%; F:75.8%), insomnia (A:81.8%; F:88.7%), abdominal bloating (A:82.2%; F:82.2%) and sexuality (A:78.8%; F:88.6%); EQ-5D to determine patients’ HRQoL (A:87.9%; F:80.3%), pain (A:87.9%; F:75.8%) and mood (A:77.7%; F:85.5%); to assess treatment adherence the Morisky-Green (A:90.9%; F:84.9%) and the dispensing register (A:80.3%; F:80.3%) were chosen. It was agreed to note in the medical record whether the patient’s treatment preferences had been considered during decision-making (A:78.8%; F:78.8%) and to use a 5-point Likert scale to assess treatment satisfaction (A:86.4%; F:86.4%). Panelists agreed (A:92.4%; F: 77.3%) to collect these PROs (1) at the time of diagnosis/relapse; (2) one month after starting treatment/change therapeutic strategy; (3) every three months during the 1^st^-year of treatment; and later (4) every six months until treatment completion/change.

**Conclusions:**

The consensus reached represents the first step towards including the patient’s perspective in AOC follow-up. The standardized collection of PROs in clinical practice may contribute to optimizing the follow-up of these patients and thus improving the quality of care.

## 1 Introduction

Ovarian cancer is the leading cause of death among all types of gynecological cancers in developed countries (1, 2). Early-stage ovarian cancer is characterized by the absence of symptoms or nonspecific symptoms associated with other less severe conditions such as minor digestive disorders or benign gynecological disorders (1, 3). Consequently, more than two-thirds of patients are diagnosed at an advanced stage, when symptoms become evident (1, 4).

Advanced ovarian cancer and its treatment cause several symptoms and substantial impact on patients’ quality of life. Knowledge of these symptoms and conditions may guide symptoms management and help monitor patients’ ability to tolerate and continue treatment (5).

Traditionally, the main goal of ovarian cancer management has been to increase lifespan, considering survival time as the common clinical endpoint. However, in recent years, there has been an increased focus on placing patients at the center of healthcare, moving towards patient-centered medicine. Patient-centered medicine aims to improve the health outcomes of individual patients in everyday clinical practice, taking into account their preferences, objectives, and values, as well as the available economic resources (6).

Currently, therapy goals for advanced ovarian cancer focus on delaying progression/recurrence, minimizing cancer-related symptoms, extension of life, and maintenance of their quality of life (7). Therefore, patient-reported outcomes (PROs) are increasingly becoming key factors in clinical decision-making within the context of advanced ovarian cancer.

A PRO is defined as any health outcome directly reported by the patient, without interpretation by physicians or other healthcare professionals (HCP) (8), and encompasses the patient’s health, quality of life, or functional status associated with healthcare or treatment (8–10). Several instruments to measure PROs, referred to as Patient-Reported Outcome Measures (PROMs), have been developed and validated. PROMs, which are generic or disease specific, are used to assess symptoms (such as nausea and vomiting, insomnia, constipation or pain), patient’s physical, social, and emotional functions, and more complex constructs such as health-related quality of life (HRQoL) (8, 10, 11).

In oncological diseases such as ovarian cancer, some studies have reported disagreement between patients’ and physicians’ perspective in terms of symptom reporting (12, 13). During patients’ follow-up, there are benefits of recording PROs, such as the improvement of symptom reporting data quality and comprehensiveness, promotion of communication between patients and clinicians, and enhancement of clinical decision making (14). PROs can be used by clinicians and researchers to measure the impact of illness and its related medical treatment on several domains of patients’ health status. The systematic and standardized collection of PROs may improve the management of oncological diseases and allow to move toward a patient-centered care. Since there is an absence of a standardized set of PROs in advanced ovarian cancer, the objective of this work is to reach a consensus on the most relevant PROs, their PROMs, and the frequency of measurement for the follow-up of advanced ovarian cancer patients from the perspective of both the patients and HCP.

## 2 Material and Methods

**Figure 1:**
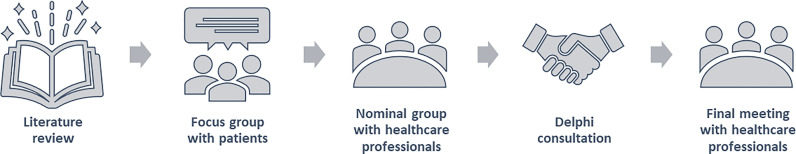
Study flow-chart.

The project comprised five steps: 1) a literature review, 2) a focus group with patients, 3) a nominal group with HCP, 4) a Delphi consultation with patients and HCP, and 5) a final meeting with HCP (**Figure 1**).

### 2.1 Literature Review

To identify PROs and PROMs in ovarian cancer, a literature review was performed by consulting the international PubMed/Medline database. Observational studies, phase III or IV clinical trials, and systematic reviews referring to the management of patients with ovarian cancer (including information on aspects of the disease and/or its treatment reported by the patient), published in English or Spanish between 07/24/2015 and 07/24/2020, were selected and reviewed (**Supplementary Material Table S1**).

In addition, the safety information collected in the technical data sheets of the main pharmacological treatments used for ovarian cancer was reviewed in order to identify other possible patient-reported symptoms related to the treatment (**Supplementary Material Table S2**).

### 2.2 Focus Group With Patients

An online focus group of patients with ovarian cancer was conducted to explore the perspective of different profiles of patients with ovarian cancer regarding the impact of the disease and its treatment on its day-to-day and assess the most relevant PROs from the patients’ perspective.

Patients were contacted and invited to participate in the focus group by the Spanish patient advocacy group of the Spanish Association of People Affected by Ovarian Cancer (*Asociación de Afectados de cáncer de ovario*, ASACO).

The PROs identified in the literature were presented, and different questions were asked for discussion: the symptoms before diagnosis, the impact of the disease and its treatment on their daily life, and their perception of the assessment of PROs during medical visits.

### 2.3 Nominal Group With Healthcare Professionals

An online nominal group meeting with HCP was held to select, based on the results of the literature review and the focus group with patients, the PROs and PROMs to be included in the Delphi consultation. PROs and PROMs were selected according to their relevance for patient follow-up and availability in the current clinical setting. Additionally, the frequency of measurement was also discussed.

The nominal group technique was used to reach a consensus on the most relevant PROs, PROMs, and frequency of measurement. The nominal group technique is a qualitative research methodology structured in four well-differentiated phases (15): 1) silent generation of ideas in writing; 2) presentation of individual ideas and clarification; 3) individual voting; and 4) presentation of individual votes and final discussion. The semi-structured group discussion ensured that all participants had the opportunity to express their ideas, favoring a balanced participation (16). The consensus was reached if ≥ 75% of the nominal group members agreed on the inclusion/exclusion of the PRO and PROM.

### 2.4 Delphi Consultation

The Delphi methodology is a widely used group survey technique for reaching consensus, typically conducted over various consecutive rounds answered anonymously by a panel of participants with relevant expertise (17). The survey rounds iteratively ask the HCP to rate the issues on implementation-related scales such as feasibility or desirability, providing controlled feedback of the previous round’s group results (18). Participants may then adjust their initial ratings based on feedback from the overall group in several subsequent iterations (19).

A two-round Delphi consultation was performed between January and March 2021. For each round, participants were given two weeks to respond to the questionnaire. Two reminders were sent to non- respondents during each period. The questionnaire of the first round consisted of two parts. In the first part, the baseline characteristics of panelists (sociodemographic information, time from diagnosis for patients; specialty and working experience information for HCP) were collected. In the second part, panelists were asked to rate a) the relevance (R) of the predefined list of PROs; b) the appropriateness (A) and feasibility (F) of the predefined PROMs for each PRO, and c) appropriateness and feasibility of two proposals for frequencies of measurement, in a nine-point Likert scale (rating scale that provides nine possible answers allowing panelists to indicate their strength of agreement regarding a topic).

The second-round questionnaire included those PROs and PROMs for which consensus was not reached and those proposed during the first round.

#### 2.4.1 Panelists

Panelists were identified and invited to participate in the Delphi consultation by the members of the nominal group, in collaboration with patient advocacy groups (ASACO) and the study coordinator.

HCP were selected based on their experience managing advanced ovarian cancer and their knowledge of PROs and PROMs. Panelists received the link of the Delphi questionnaire, username, and password (exclusive for each participant) by e-mail.

#### 2.4.2 Consensus Definition

The consensus was reached for each PRO or PROM and its frequency of measurement when at least 75% of the panelists rated it as ‘relevant’, ‘appropriate’, or ‘feasible’ (score 7-9). The definition of consensus was established before data analyses, according to the standard criteria (19).

#### 2.4.3 Data Analysis

The percentage of panelists who selected each option and percentile distributions (25, 50, and 75) were calculated using STATA statistical software, V.14. The percentages described in the text refer to the final scores (score of the round in which consensus was achieved).

### 2.5 Final Meeting

A final meeting with the members of the nominal group was conducted to review the results of the Delphi consultation and define the final set of PROs and PROMs.

During this meeting, the barriers that could hinder the collection of PROs in clinical practice were also explored.

## 3 Results

### 3.1 Literature Review

The search yielded 56 references potentially relevant, of which 13 were considered eligible for inclusion. One additional publication was identified during the search of key reference list. The revision of these 14 publications identified 28 PROs and 15 PROMs. Vomiting (57.1%), fatigue (57.1%), nausea (57.1%), and diarrhea (50.0%) were assessed in more than half of the studies reviewed. The HRQoL questionnaires developed by the European Organisation for Research and Treatment of Cancer (EORT-QLQ-c30 and EORT-QLQ-OV28) and by the Functional Assessment of Cancer Therapy-Endocrine Symptoms (FACT-O), and the generic HRQoL questionnaire EuroQoL (EQ-5D), were the most frequently used PROMs.

Additionally, the review of the technical data sheets of treatments identified 44 patient-reported symptoms related to the treatment. Skin disorders (93.7% of technical data sheets), vomiting (93.7%), nausea (93.7%), diarrhea (87.5%), alopecia (75.0%), asthenia (75.0%), abdominal pain (75.0%), and anorexia (75.0%) were the most frequently identified PROs appearing in the technical data sheets.

### 3.2 Focus Group With Patients

Five patients with ovarian cancer (age range: 36-67 years; time from diagnosis range: 19 months to 7 years; 80% diagnosed at stage III; 80% had received treatment for ovarian cancer) participated in the focus group.

Patients reported that before diagnosis non-specific pain, fatigue and anxiety were the symptoms most frequently perceived. However, once the treatment started, the main symptomatology experienced by patients was extreme tiredness and fatigue, pain, insomnia, mood swings/disturbance (including depression and anxiety), difficulty in accepting the illness, and sadness. Regarding the specific symptoms faced during the treatment period, the participants highlighted fatigue (physical exhaustion) and lack of energy, as well as psychological and emotional distress, digestive discomfort (nausea and vomiting), skin problems (dry skin), alopecia (with impact on body image) and sexuality problems (vaginal dryness and decreased libido).

Regarding assessing the PROs in clinical practice, the patients agreed that some of these PROs were evaluated during follow-up; however this was done informally without using specific questionnaires for this purpose.

### 3.3 Nominal Group of Healthcare Professionals

Twelve hospital pharmacists and four oncologists participated in the nominal group. The PROs and PROMs identified in the literature review and the focus group with patients were presented during the nominal group.

HCP assessed the relevance of PROs related to symptoms (grouped into 17 categories: fatigue, pain, sexuality, body image, eating disorders, cognitive capacity, dermatological diseases, mood, insomnia, gastrointestinal disorders, respiratory system disorders, neuropathy, hypersensitivity, oral cavity disorders, palpitations, dizziness, and chills) and those related to more complex constructs such as HRQoL, functional status, social function, preferences, adherence, and satisfaction. Later, the most adequate and feasible PROMs and measurement frequency to be used in clinical practice were also discussed during the nominal group.

The PROs and PROMs that the nominal group agreed to include in the Delphi consultation are shown in **Table 1**. Two frequencies of measurement were proposed by the nominal group: 1) at diagnosis; every 3-4 months during the first two years; every six months from the 3^rd^ to the 5th year, and later annually; or 2) at diagnosis; one month after starting treatment/change in therapeutic strategy; every three months during the first year of treatment; and later every six months until completion or change of treatment.

**Table 1:** PROs and PROMs agreed upon by the nominal group, presented in the Delphi survey.

PROs	PROMs
Constipation	PRO-CTCAE
Diarrhea	PRO-CTCAE
Nausea	PRO-CTCAE
Gastrointestinal disorders*	EORTC-QLQ-OV28*^†^*
Fatigue	PRO-CTCAE
Mood	EORTC-QLQ-OV28*^†^* EQ-5D PRO-CTCAE
Pain	EORTC-QLQ-OV28*^†^* EQ-5D PRO-CTCAE
Sexuality	EORTC-QLQ-OV28*^†^* PRO-CTCAE
Neuropathy	EORTC-QLQ-OV28*^†^* PRO-CTCAE
Insomnia	PRO-CTCAE
HRQoL	EQ-5D (VAS)
Adherence	VAS (0=non adherent; 10=completely adherent) Morisky-Green Dispensing register
Preferences	to note in the medical record whether the patient’s preferences had been considered during decision-making
Satisfaction	5-point Likert scale

EORTC-QLQ-OV28, European Organization for Research and Treatment of Cancer Quality of Life Questionnaire - Ovarian Cancer Module; EQ-5D, EuroQoL quality of life questionnaire; HRQoL, Health Related Quality of Life; PRO-CTCAE, Patient-Reported Oucomes version of the Common Terminology Criteria for Adverse Events; VAS, Visual Analogue Scale; ^†^The EORTC-QLQ-OV28 questionnaire collects information on pain, gastrointestinal disorders, neuropathy, sexuality, and mood/concern with future health; *including constipation, diarrhea and nausea.

### 3.4 Delphi Consultation

A total of 56 HCP experts in the management of advanced ovarian cancer (51.8% Hospital Pharmacy; 41.1% Oncology; 3.6% Nursing; and 3.6% Psycho-oncology; mean time in specialty 12.5 [SD: 8.0] years) and ten patients with advanced ovarian cancer (mean age: 50.8 years [SD: 8.9]; mean time from diagnosis 5.4 [SD: 3.0] years) participated in the first round of the Delphi consultation. The response rate of the second round was 96.4% (n=54) in professionals and 80% (n=8) in patients.

Results of the Delphi consultation are shown in **Supplementary Material Table S3**. All PROs presented in the Delphi questionnaire reached consensus regarding their relevance for the follow-up of patients with advanced ovarian cancer: adherence (95.5%), fatigue (95.5%), HRQoL (95.5%), neuropathy (95.5%), pain (93.9%), constipation (90.9%), satisfaction with treatment (90.9%), nausea (90.1%), diarrhea (89.4%), mood (87.9%), preferences (84.9%), insomnia (78.8%) and sexuality (78.8%). During the first round, panelists proposed two additional PROs: skin dryness and abdominal bloating. These additional PROs were included in the second-round questionnaire; however, consensus on relevance was only achieved for abdominal bloating (87.1%).

Agreement was reached for the Patient-Reported Outcomes version of the Common Terminology Criteria for Adverse Events (PRO-CTCAE™) to be used to collect data on abdominal bloating (A:82.2%; F:82.2%), constipation (A:86.4%; F:75.8%), diarrhea (A:87.9%; F:88.7%), fatigue (A:84.9%; F:75.8%), insomnia (A:81.8%; F:88.7%), mood (A:80.3%; F:88.6%), nausea (A:89.4%; F:75.8%), neuropathy (A:92.4%; F:77.3%), pain (A:89,4; F: 78,8) and sexuality (A:78.8%; F:88.6%). The PRO-CTCAE Measurement System characterizes the frequency, severity, interference, and presence/absence of symptomatic toxicities that can be meaningfully reported from the patient’s perspective. The National Cancer Institute (USA) developed it to improve the validity, reliability, and precision with which symptomatic adverse effects of treatment are evaluated in patients participating in cancer clinical trials (20).

It was agreed to use the EQ-5D (21) to collect HRQoL. EQ-5D is an HRQoL generic questionnaire developed by the EuroQoL Group. This questionnaire comprises five dimensions: mobility, self-care, usual activities, pain and discomfort, and anxiety and depression. Additionally, it includes a standard vertical 20-cm visual analogue scale that assesses overall health on the day that the respondent completes the questionnaire (21). Since EQ-5D also assess pain and discomfort, panelists agreed to use this questionnaire to collect mood (A:77.7%; F:85.5%) and pain (A:87.9%; F:75.8%).

To assess adherence, panelists agreed to use the Morisky-Green 4-item scale (A:90.9%; F:84.9%) and to review the dispensing register (A:80.3%; F:80.3%) to complement patient-reported data. Morisky-Green 4-items scale is an easy, validated, generic self-reported, medication-taking behavior tool (22).

It was agreed to register in the medical record the patient’s preferences during the decision-making process (A:78.8%; F:78.8%) and to use a 5-point Likert scale to assess satisfaction with treatment (A:86.4%; F:86.4%).

Regarding the frequency of measurement, panelists reached a consensus to assess PROs (A: 92.4%; F: 77.3%): (1) at diagnosis; (2) one month after starting treatment/change in therapeutic strategy; (3) every three months during the first year of treatment; and later (4) every six months until completion or change of treatment.

### 3.5 Final Meeting

The members of the nominal group reviewed the results of the Delphi consultation. For those PROs where consensus was reached on the use of more than one PROM, members of the nominal group debated the suitability and feasibility of using both PROMs or only one of them.

Thus, according to the results of the Delphi consultation, agreement was reached on the use of PRO-CTCAE and EQ-5D to collect data on pain and mood. During the final meeting, it was considered that the information gathered by both PROMs was similar and, therefore, they agreed to use only the EQ-5D to assess pain and mood.

**Figure 2:**
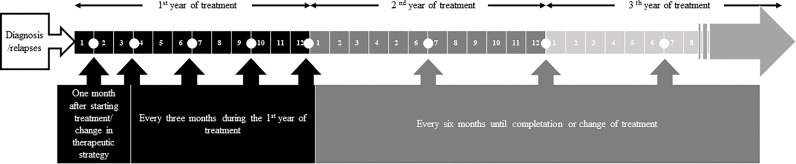
Frequency of PROs collection in clinical practice.

Similarly, panelists of the Delphi consultation reached a consensus on using the Morisky-Green 4-item scale (22) and the dispensing register to evaluate patient adherence to treatment. In this case, members of the nominal group indicated that instruments provided complementary information, and therefore, they agreed to use both. **Table 2** and **Figure 2** show the final set of PROs and PROMs.

**Table 2:** Final set of PROs and PROMs.

PRO	PROM
**Fatigue**	PRO-CTCAE
**Neuropathy**	PRO-CTCAE
**Diarrhea**	PRO-CTCAE
**Constipation**	PRO-CTCAE
**Nausea**	PRO-CTCAE
**Insomnia**	PRO-CTCAE
**Abdominal bloating**	PRO-CTCAE
**Sexuality**	PRO-CTCAE
**Pain**	EQ-5D
**Mood**	EQ-5D
**HRQoL**	EQ-5D
**Preferences**	Medical record
**Adherence**	Morisky Green and dispensing register
**Satisfaction**	5-point Likert scale

EQ-5D, EuroQoL quality of life questionnaire; HRQoL, Health Related Quality of Life; PRO-CTCAE, Patient-Reported Oucomes version of the Common Terminology Criteria for Adverse Events.

Related to the frequency of measurement, the members of the nominal group pointed out the importance of collecting PROS at diagnosis and relapses, therefore, relapse was included on frequency of measurement as follow: (1) at diagnosis/relapse; (2) one month after starting treatment/change in therapeutic strategy; (3) every three months during the first year of treatment; and later (4) every six months until completion or change of treatment.

Additionally, during this meeting identified several barriers that should be addressed to promote and ensure the collection of PROs in clinical practices were identified: 1) barriers related to the health system itself; 2) barriers associated with healthcare professionals; and 3) barriers related to patients. Experts pointed out that the lack of resources (i.e., electronic register, questionnaires, and professional staff) and the heterogeneity of the healthcare processes, organizational models as well as information systems within different regions may hinder the use of PROs in clinical practice. The limited education and information of patients and healthcare professionals about PROMs have also been identified as barriers to implementing the set of PROs and PROMs defined.

## 4 Discussion

During the past decades, it has been recognized that the evaluation of symptoms, functional status, and overall HRQoL from the patient’s perspective is crucial to providing optimal healthcare. Therefore, the definition of a set of PROs and PROMs to be assessed during the follow-up of patients with advanced ovarian cancer represents an important step towards the inclusion of the patient’s perspective in managing the disease.

The standardized collection of PROs in clinical practice may contribute to optimizing the follow-up of these patients and thus improving the quality of care they receive. Patients have various perspectives about living with a condition, which may differ from those of clinicians and researchers (14). Incorporating the patient’s perspective in clinical practice, encouraging patient participation in decision-making and self-management is critical to ensure that measured outcomes reflect those considered most relevant by patients (23). Standard sets of health outcomes, including clinical PROs, have been developed for other oncological diseases. However, none of these initiatives have focused on ovarian cancer.

Patients and healthcare professionals agreed on the relevance of measuring 14 PROs during advanced ovarian cancer follow-up. Monitoring of the following patient-reported symptoms associated with the disease and its treatment were considered essential: fatigue, neuropathy, diarrhea, constipation, nausea, insomnia, abdominal bloating, sexuality, and pain were considered. In addition, assessment of mood disturbance, HRQoL, patients’ preferences regarding treatment characteristics, treatment adherence, and treatment satisfaction were identified as key aspects to consider for disease management.

Several initiatives have been developed to identify a core set of PROs in patients with cancer that should be measured in clinical trials (5, 24). In line with our results, abdominal pain, bloating, cramping, fear of recurrence/disease progression, indigestion, sexual dysfunction, vomiting, weight gain, and weight loss were identified PROs specific to ovarian cancer (5). Anorexia, cognitive problems, constipation, diarrhea, dyspnea, fatigue, nausea, neuropathy, pain, and insomnia were indicated as the most important PROs across cancer types (5).

PROs are typically assessed using PROMs, and many validated questionnaires are available for ovarian cancer (5). The selection of the PROMs to be applied was based on their appropriateness and feasibility in clinical practice. To determine the appropriateness of the PROM, content validity, construct validity and reliability were considered. To assess feasibility, practical considerations regarding cost, burden, language availability, mode of administration, length, among others were taken into account. Consensual PROMs, PRO-CTCAE™, EQ-5D, and Morisky-Green, are widely used in patients subject to cancer monitoring.

The use of PROMs continues to expand beyond clinical research in recognition of the potential of this information to improve the quality of healthcare by placing the patient at the center of decision making; however, their widespread use and feasibility have been limited by several barriers. Consistent with previous studies (25), barriers related to healthcare systems, healthcare professionals, and patients have been identified. Most obstacles are inherent to the structure of the Spanish national healthcare system, namely the heterogeneity of this system, including the healthcare process, organization models and information systems, and the lack of resources. The lack of digital tools allowing systematic and automatic PROMs compilation has also been identified as one of the main barriers to be tackled to ensure the monitoring of the set of PROs. The availability of electronic tools and adequate technology to support PROMs collection in clinical practice would reduce the burden in terms of the consultation schedule (26, 27). Additionally, the development of education and information programs about PROs and PROMs addressed to patients and healthcare professionals may promote PROs collection in clinical practice (26–28).

This project presents several limitations inherent to its design. First, this set of PROs and PROMs reflects the opinion of a multidisciplinary group of 72 healthcare professionals involved in the management of ovarian cancer and 15 patients. Although no significant differences are expected, different participants could have reached a consensus on other PROs and PROMs. Some details such as type of treatment or duration of treatment of focus group participants were not collected; nevertheless, it is expected that given the different time from diagnosis and the stage of the disease, the main available therapies would be represented. The PROs and PROMs selected reflect current therapeutic strategies but as treatments for advanced ovarian cancer continue to develop, the nature of the symptoms and their impact on patients’ HRQoL may also vary. With the incorporation of new agents in the range of therapeutic options for ovarian cancer, regular updates on the set defined is recommendable. Although a multidisciplinary team, including both healthcare professionals involved in managing ovarian cancer and patients participated in the study, it was confined to the Spanish setting. Therefore, some of the selected PROs may only be relevant for Spanish patients. However, to facilitate the extrapolation of the project results to other settings, the selected PROMs are available in different languages and are not specific to the Spanish population. Finally, the recent COVID-19 pandemic made face-to-face focus groups and nominal groups facilitation untenable. Despite this, online meetings (focus group and nominal group) have the potential to recruit demographically and geographically diverse participants. Conducting the meetings online is not expected to impact the project results.

Despite these limitations, this work highlights the value of taking into account both clinicians’ and patients’ perspectives when developing interventions to improve the quality of care (29, 30). This wider perspective could reduce the observed discrepancies between clinicians and patients on disease assessment, treatment preferences, or factors to be considered in decision-making (23).

## 5 Conclusion

This is the first study determining a set of PROs and PROMs in advanced ovarian cancer, considering the perspective of both healthcare professionals and patients. The standardized collection of PROs in advanced ovarian cancer is a starting point to improving the quality of care. In addition, identifying possible barriers to subsequent implementation may help define optimized strategies to foster its use in clinical practice.

## Data Availability Statement

The original contributions presented in the study are included in the article/**Supplementary Material**. Further inquiries can be directed to the corresponding author.

## Author Contributions

VE-V, EB, GC, RC-B, RD-F, AFR, CF, LG-C, MH-F, GM, CMN, MR, AR, PS-F, MVC and EG contributed equally to this work with data acquisition and data interpretation. MC contributed to the conceptualizaation and design of the study and wrote the first draft of the manuscript. All authors contributed to manuscript revision, read and approved the submitted version.

## Funding

The project was sponsored by GlaxoSmithKline. The funder was not involved in the study design, collection, analysis, interpretation of data, the writing of this article or the decision to submit it for publication.

## Conflict of Interest

VE-V received support to continuing education/advisory fees from Astellas, Bristol-Myers Squibb, GlaxoSmithKline, Janssen, Merck Sharp & Dohme, Novartis, Roche, and Sanofi. RC-B has received support to continuing education/advisory fees: Boehringer-Ingelheim, Janssen, Merck Sharp & Dohme, Hoffmann-La Roche, Amgen Inc, GlaxoSmithKline, and Pfizer. EG has received advisory/consultancy honorarium from AstraZeneca-MSD, Clovis Oncology, GSK-Tesaro, PharmaMar, Roche; has received speaker bureau/expert testimony honorarium from AstraZeneca-MSD, PharmaMar, Roche, GSK-Tesaro, Clovis; and has received travel/accommodation/expenses from Roche, GSK-Tesaro and Baxter. MV works for an independent research entity that received funding from GlaxoSmithKline to coordinate and conduct the study.

The remaining authors declare that the research was conducted in the absence of any commercial or financial relationships that could be construed as a potential conflict of interest.

## Publisher’s Note

All claims expressed in this article are solely those of the authors and do not necessarily represent those of their affiliated organizations, or those of the publisher, the editors and the reviewers. Any product that may be evaluated in this article, or claim that may be made by its manufacturer, is not guaranteed or endorsed by the publisher.
