# Supplementary material for: Defining a Standard Set of Patient-Reported Outcomes for Patients With Advanced Ovarian Cancer
Source: Front Oncol. 2022 May 18;12:885910. doi: 10.3389/fonc.2022.885910 (PMC9159390; doi:10.3389/fonc.2022.885910)
Supplement: Supplementary file 1 [file Table_1.docx]

Supplementary Table S1: Search terms and strategy in Medline/Pubmed

| Database | Pubmed/Medline |
| --- | --- |
| **Search period** | July, 24th 2015- July, 24th 2020 (beginning of the project) |
| **Search strategy** | ("Carcinoma, Ovarian Epithelial"[Mesh] OR Epithelial ovarian cancer [tiab] OR Advanced ovarian cancer [tiab]) AND ("Quality of Life"[Mesh] OR Health-Related Quality Of Life [tiab] OR patient reported outcome*[tiab] OR patient related outcome*[tiab] OR patient-reported outcome*[tiab] OR patient-related outcome*[tiab] OR patient reported outcome*[ot] OR patient related outcome*[ot] OR patient-reported outcome*[ot] OR patient-related outcome*[ot] OR “Patient Satisfaction”[Mesh] OR “Patient Preference”[Mesh] OR “Patient compliance”[Mesh] OR “Medication adherence”[Mesh] OR Compliance |
|  |  |

* = truncation character (wildcard); [tiab] = limit to title or abstract
