# Supplementary material for: Defining a Standard Set of Patient-Reported Outcomes for Patients With Advanced Ovarian Cancer
Source: Front Oncol. 2022 May 18;12:885910. doi: 10.3389/fonc.2022.885910 (PMC9159390; doi:10.3389/fonc.2022.885910)
Supplement: Supplementary file 2 [file Table_2.docx]

Supplementary Table S2: List of technical data sheets of treatments reviewed

| Anastrozole |
| --- |
| Bevacizumab |
| Carboplatin |
| Cisplatin |
| Cyclophosphamide |
| Docetaxel |
| Etoposide |
| Exemestane |
| Gemcitabine |
| Letrozole |
| Niraparib |
| Olaparib |
| Paclitaxel |
| Pegylated liposomal doxorubicin |
| Rucaparib |
| Tamoxifen |
| Topotecan |
| Trabectedin |
