# Supplementary material for: Defining a Standard Set of Patient-Reported Outcomes for Patients With Advanced Ovarian Cancer
Source: Front Oncol. 2022 May 18;12:885910. doi: 10.3389/fonc.2022.885910 (PMC9159390; doi:10.3389/fonc.2022.885910)
Supplement: Supplementary file 3 [file Table_3.docx]

Supplementary Table S3: Results of the Delphi round consultation

| **PRO** | **Relevance consensus**  **Round 1** | | | PROM | **Appropriate consensus**  **Round 1** | | | **Feasible consensus**  **Round 1** | | | **Appropriate consensus**  **Round 2** | | | **Feasible consensus**  **Round 2** | | |
| --- | --- | --- | --- | --- | --- | --- | --- | --- | --- | --- | --- | --- | --- | --- | --- | --- |
|  | **%** | **Mean (SD)** | **P75,P50,P25** |  | **%** | **Mean (SD)** | **P75,P50,P25** | **%** | **Mean (SD)** | **P75,P50,P25** | **%** | **Mean (SD)** | **P75,P50,P25** | **%** | **Mean (SD)** | **P75,P50,P25** |
| **Symptoms (pain, GI disorders, neuropathy, sexuality, mood)** | - | - | - | **EORTC-QLQ-OV28†** | **95.5** | 8.0 (1.1) | 9,8,8 | *65.2* | *6.9 (1.9)* | *8,7,6* | - | - | - | - | - | - |
| **Pain** | **93.9** | 8.5 (1.2) | 9,9,8 | **EQ-5D** | **87.9** | 7.7 (1.4) | 9,8,7 | **75.8** | 7.4 (1.7) | 9,8,7 | - | - | - | - | - | - |
|  |  |  |  | **PRO-CTCAE** | **89.4** | 7.8 (1.4) | 9,8,8 | **78.8** | 7.5 (1.6) | 9,8,7 | - | - | - | - | - | - |
| **Fatigue** | **95.5** | 8.3 (0.9) | 9,9,8 | **PRO-CTCAE** | **84.9** | 7.6 (1.5) | 9,8,7 | **75.8** | 7.2 (1.9) | 8.7,8,7 | - | - | - | - | - | - |
| **Neuropathy** | **95.5** | 8.3 (0.9) | 9,9,8 | **PRO-CTCAE** | **92.4** | 7.9 (1.2) | 9,8,7 | **77.3** | 7.5 (1.5) | 9,8,7 | - | - | - | - | - | - |
| **GI disorders‡** | **87.9** | 7.9 (1.5) | 9,8,8 | **‡** | - | - | - | - | - | - | - | - | - | - | - | - |
| **Diarrhea** | **89.4** | 7.9 (1.4) | 9,8,7 | **PRO-CTCAE** | **87.9** | 7.7 (1.4) | 9,8,7 | *72.7* | *7.2 (1.8)* | *9,8,6* | - | - | - | **88.7** | 7.9 (1.3) | 9,8,7.7 |
| **Constipation** | **90.9** | 8.1 (1.2) | 9,9,7 | **PRO-CTCAE** | **86.4** | 7.6 (1.7) | 9,8,7 | **75.8** | 7.2 (2.0) | 8.7,8,7 | - | - | - | - | - | - |
| **Nauseas** | **90.1** | 8.1 (1.2) | 9,8,8 | **PRO-CTCAE** | **89.4** | 7.7 (1.6) | 9,8,7 | **75.8** | 7.2 (2.0) | 9,8,7 | - | - | - | - | - | - |
| **Insomnia** | **78.8** | 7.3 (1.4) | 8,7,7 | **PRO-CTCAE** | **81.8** | 7.3 (1.8) | 8,8,7 | *71.2* | *7.0 (2.1)* | *8.7,8,6* | - | - | - | **88.7** | 7.6 (1.5) | 9,8,7 |
| **Mood** | **87.9** | 7.9 (1.3) | 9,8,7 | **EQ-5D** | **77.7** | 7.2 (1.6) | 8,8,7 | *68.2* | *7.1 (1.9)* | *9,8,6* | - | - | - | **85.5** | 7.6 (1.4) | 9,8,7 |
|  |  |  |  | **PRO-CTCAE** | **80.3** | 7.3 (1.7) | 8,8,7 | *72.7* | *7.1 (1.8)* | *8,8,6* | - | - | - | **88.6** | 7.5 (1.4) | 8,8,7 |
| **Sexuality** | **78.8** | 7.3 (1.6) | 8,7,7 | **PRO-CTCAE** | **78.8** | 7.3 (1.7) | 8,8,7 | *72.7* | *7.0 (1.8)* | *8,8,6* | - | - | - | **88.6** | 7.4 (1.5) | 8,8,7 |
| **HRQoL** | **95.5** | 8.4 (0.9) | 9,9,8 | **EQ-5D (VAS)** | **87.9** | 7.7 (1.5) | 8,8,7 | **80.3** | 7.7 (1.9) | 9,8,7 | - | - | - | - | - | - |
| **Preferences** | **84.9** | 7.7 (1.2) | 8,8,7 | **Medical record** | **78.8** | 7.7 (1.4) | 9,8,7 | **78.8** | 7.6 (1.6) | 9,8,7 | - | - | - | - | - | - |
| **Adherence** | **95.5** | 8.5 (0.9) | 9,9,8 | **Morinsky Green** | **90.9** | 7.9 (1.3) | 9,8,7 | **84.9** | 7.8 (1.5) | 9,8,7 |  |  |  |  |  |  |
|  |  |  |  | **VAS (0=non adherent; 10=completely adherent)** | *59.1* | *6.7 (1.7)* | *8,7,6* | *77.3* | *7.4 (1.8)* | *9,8,7* |  |  |  |  |  |  |
|  |  |  |  | **Dispensing register** | **80.3** | 7.5 (1.7) | 9,8,7 | **80.3** | 7.7 (1.7) | 9,8,7 |  |  |  |  |  |  |
| **Satisfaction** | **90.9** | 7.9 (1.2) | 9,8,8 | **5 point- Likert scale** | **86.4** | 7.7 (1.4) | 9,8,7 | **86.4** | 7.8 (1.7) | 9,8,8 |  |  |  |  |  |  |
| **Abdominal bloating^α^** | **87.1** | 7.8 (1.3) | 9,8,8 | **PRO-CTCAE** |  |  |  |  |  |  | **82.2** | 7.5 (1.4) | 8,8,7 | **82.2** | 7.5 (1.6) | 8,8,7 |
| **Dry skin^α^** | *58.0* | *6.6 (1.5)* | *8,7,5.7* | **PRO-CTCAE** |  |  |  |  |  |  | 71.0 | 7.0 (1.4) | 8,7,6 | 72.5 | 7.1 (1.6) | 8,7,6 |
| **Follow-up frequency 1*** |  | - | - | ***** | *68.2* | *6.8 (2.0)* | *8,7,6* | *65.2* | *6.9 (1.9)* | *8.7,7,6* |  |  |  |  |  |  |
| **Follow-up frequency 2**** |  | - | - | ****** | **92.4** | 8.0 (1.0) | 9,8,8 | **77.3** | 7.4 (1.5) | 9,8,7 |  |  |  |  |  |  |

*SD: Standard deviation; EORTC-QLQ-OV28: European Organization for Research and Treatment of Cancer Quality of Life Questionnaire - Ovarian Cancer Module; EQ-5D: EuroQoL quality of life questionnaire; VAS: Visual Analogue Scale; GI: gastrointestinal; P25: percentile 25; P50: percentile 50; P75: percentile 75; PRO: Patient Reported Outcomes; PRO-CTCAE: Patient-Reported Outcomes Version of the Common Terminology Criteria for Adverse Events.*

*†The EORTC-QLQ-OV28 questionnaire collects information on pain, gastrointestinal disorders, neuropathy, sexuality, and mood / concern with future health;*

*‡* *Assessment of gastrointestinal disorders in general would be included in the administration of the EORTC-QLQ-OV28. The assessment of gastrointestinal disorders was presented using instruments for collecting specific PROs for each symptom individually.*

*^α^Assessed in the second round of Delphi consultation*

**Follow-up 1: (1) at the time of diagnosis; (2) year 1 and 2 after diagnosis: every 3 or 4 months; (3) years 3 to 5 after diagnosis: every 6 months; (4) from year 5 after diagnosis: annual.*

***Follow-up 2: (1) at the time of diagnosis/relapse; (2) one month after starting treatment/change in therapeutic strategy; (3) every three months during the first year of treatment; and later (4) every six months until completion or change of treatment.*
